# Heating Up to Heal—Acute Heat Exposure Increases Hepatic Mitophagy Resulting in Hormetic Improvements in Mitochondrial Bioenergetic Efficiency

**DOI:** 10.1093/function/zqab011

**Published:** 2021-02-22

**Authors:** Kelsey H Fisher-Wellman

**Affiliations:** 1 Department of Physiology, East Carolina University, Brody School of Medicine, Greenville, NC 27834, USA; 2 East Carolina Diabetes and Obesity Institute, Greenville, NC 27834, USA

## A Perspective on “Heat treatment improves hepatic mitochondrial respiratory efficiency via mitochondrial remodeling.”

In the latest issue of *Function*, Von Schulze et al.^1^ elegantly combine metabolic physiology with sophisticated bioenergetic analyses to elucidate the acute and chronic impacts of heat treatment (HT) across the hepatic mitochondrial network. This work is based on a collection of evidence, from this group and others, suggesting that impairments in mitochondrial functionality and/or quality control may underly non-alcoholic fatty liver disease (NAFLD).^2–4^ Recently, in high-fat-fed rats, this group demonstrated an improvement in whole-body glucose tolerance upon exposure to weekly HT.^5^ In that study, expression of heat shock protein 72 (HSP72) tracked with systemic metabolic improvements, and siRNA knockdown of HSP72 in primary hepatocytes increased lipid droplet formation, presumably due to disruptions in mitochondrial network integrity. This study was designed to address two primary questions: (1) are the metabolic benefits induced by HT due to intrinsic improvements in hepatic mitochondrial quality and efficiency and (2) is HSP72 required for these effects. Deciphering the latter was made possible by leveraging a first-in-class genetic mouse model of liver-specific HSP72 deficiency.

With respect to mitochondrial bioenergetics, using multiple mouse cohorts (wild type and HSP72 KO; KO) and diet interventions (low-fat diet [LFD] vs. high-fat diet [HFD]), they convincingly demonstrate that short-term heat exposure acutely lowers mitochondrial respiratory efficiency, reflected as an increase in the ratio of peroxide emission to oxygen consumption (*J*H_2_O_2_/*J*O_2_, also known as “electron leak”). Importantly, as a beautiful example of linking *in vitro* biochemical flux analysis to organism physiology, hepatic mitochondrial inefficiency occurred alongside systemic increases in serum lactate, the predictable consequence of acute mitochondrial disruption. As for the cause of respiratory insufficiency induced by acute HT, immediately following exposure, increases in mitochondrial fragmentation were noted, as was mitophagy flux (assessed via Western blotting and confocal microscopy). Interestingly, while mitochondrial respiratory efficiency was restored as early as 1-h post HT, a variety of mitophagy markers remained elevated at the 2-h time-point, the most notable of which was mitochondrial ubiquitination. Similar discordance between mitochondrial efficiency and the transcriptome was apparent in RNA-seq data comparing 0–24-h HT, further highlighting the critical importance of functionally characterizing the mitochondrial network.

Although a single exposure to HT acutely lowered mitochondrial efficiency, repeated exposures in both LFD and HFD cohorts resulted in adaptive increases across the hepatic mitochondrial network that both improved respiratory kinetics and lowered electron leak. Such changes were associated with systemic metabolic improvements in blood glucose, as well as a trend for lower liver triglycerides. Surprisingly, despite strong associations linking increased HSP72 to less severe NAFLD,^6^^,^^7^ using a first-in-class genetic mouse model of HSP72 deficiency, the authors provide clear evidence that HT-induced mitochondrial remodeling in the liver does not require HSP72. Interestingly, however, the ability of HT to lower blood glucose was absent in HSP72 KO mice fed an HFD, suggesting that HSP72 may mediate glucose homeostasis, independent of hepatic mitochondrial bioenergetics. Taken together, their findings indicate that HT promotes acute hepatic heat stress that temporarily reduces mitochondrial efficiency. Such changes coincide with network fragmentation and increased mitophagy that rapidly restores respiratory kinetics in the short-term and improves hepatic mitochondrial efficiency in the long-term and associates with improvements in systemic metabolism.

**Figure 1.**
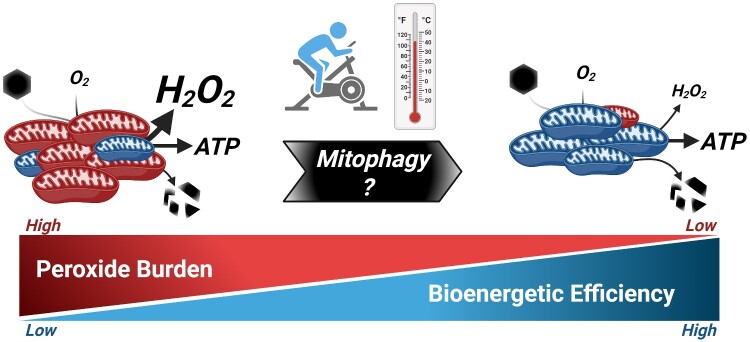
Model Whereby the Induction of Mitophagy Improves Intrinsic Mitochondrial Bioenergetic Efficiency. Theoretical model in which repeated exposures to exercise and HT interventions activate mitophagy flux that shifts the mitochondrial network in favor of a smaller, more efficient mitochondrial network that minimizes cellular peroxide burden.

From a therapeutic perspective, the results of Von Schulze et al.^1^ have far-reaching implications, particularly with respect to individuals suffering from NAFLD for which exercise interventions are contraindicated. With respect to hepatic mitochondrial biology, one of the most interesting findings of this work centers upon the observation that chronic HT both improved intrinsic bioenergetic efficiency while simultaneously decreasing hepatic mitochondrial content. Such findings raise the intriguing possibility that, at least for hepatic metabolic health, mitochondrial quality may be more important than content. While speculative, it is possible that intrinsic bioenergetic efficiency exists on a continuum within the hepatocyte mitochondrial network from highly efficient to inefficient units. Although the biological implications remain unresolved, metabolic heterogeneity is well documented both within and between mammalian cells, most notably in various cancer model systems.^8^^,^^9^ In addition to oxidative ATP synthesis (ie, OXPHOS), mitochondria across the body contribute to a variety of energetic and biosynthetic outputs. While distinct combinations of mitochondrial quantity and quality may be capable of meeting identical “output” demands, a decrease in mitochondrial quality may result in a subpopulation of inefficient mitochondria that drives pathology for example by elevating cellular peroxide burden (Figure 1). Such inefficiency may derive from the respiratory complexes, as Complexes I and II (CI and CII) were found to be rapidly degraded in response to acute heat stress in this study and may be similarly susceptible to generalized proteotoxic stressors. Both CI and CII are well-documented sources of mitochondrial electron leak.^10^ Thus, interventions that trigger an increase in mitophagy (eg, exercise or HT) may improve organ health by accelerating the degradation of “damaged” and/or inefficient respiratory complexes that in turn maximizes bioenergetic efficiency across the network (Figure 1). Moving forward, as technological advancements allow for functional characterization of mitochondrial heterogeneity, it will be interesting to observe how such changes relate to organ and whole-body metabolic health.

In conclusion, the work of Von Schulze et al. provides an elegant “blue print” for integrating physiological outcomes to the underlying mitochondrial bioenergetics. Although future work will be required to definitively tease out the role of increased mitophagy in NAFLD, the present findings provide strong basic science rationale for therapeutic interventions involving HT. Moreover, the novel HSP72 mouse model provides an interesting opportunity to explore tissue-specific differences in the heat shock response attributable to HSP72.

## Funding

The work was supported by DOD-W81XWH-19-1-0213.

## Conflict of Interest Statement

None declared.
